# Paralytic rabies in a goat

**DOI:** 10.1186/s12917-018-1681-z

**Published:** 2018-11-12

**Authors:** Igor Louzada Moreira, Davi Emanuel Ribeiro de Sousa, Jair Alves Ferreira-Junior, Márcio Botelho de Castro, Tayná Cardim Morais Fino, José Renato Junqueira Borges, Benito Soto-Blanco, Antonio Carlos Lopes Câmara

**Affiliations:** 1 Large Animal Veterinary Teaching Hospital, College of Agronomy and Veterinary Medicine, Universidade de Brasília, Galpão 4, Granja do Torto, Brasília, DF 70636-200 Brazil; 2 Veterinary Pathology Laboratory, College of Agronomy and Veterinary Medicine, Universidade de Brasília, Via L4 Norte s/n, Asa Norte, Brasília, DF 70910-970 Brazil; 3 Department of Veterinary Clinics and Surgery, Veterinary College, Universidade Federal de Minas Gerais, Avenida Presidente Antônio Carlos 6627, Belo Horizonte, MG 31275-013 Brazil

**Keywords:** Central nervous system, Immunohistochemistry, *Lyssavirus*, *Rhabdoviridae*, Viral diseases

## Abstract

**Background:**

Paralytic form of rabies is frequent in cattle in Latin America, but it is uncommon in goats. There are few clinical reports on furious rabies affecting goats, and the sporadic cases of rabid goats from surveillance programs worldwide lack clinical data. Furthermore, few studies reported the cerebrospinal fluid findings in rabid livestock.

**Case presentation:**

On a farm in Midwestern Brazil, six of 47 Saanen goats died within one week. No vaccination protocols were implemented on the farm and the owner stated bat bites history on the livestock. Although rabies is endemic in Brazil, livestock vaccination is not mandatory. One 1-year-old buck was evaluated and showed non-specific clinical signs evolving within 12-h to nervous signs. Cerebrospinal fluid analysis revealed mononuclear pleocytosis, hyperproteinemia and high glucose levels. At necropsy, no gross lesions were present. Microscopically, discrete to moderate perivascular lymphoplasmacytic cuffing in gray and white matter, neuronal necrosis, neuronophagia, and mononuclear ganglioneuritis was observed in the brainstem and cervical spinal cord. Immunohistochemistry revealed strong anti-rabies virus immunostaining. Fresh central nervous system samples were positive for rabies in direct fluorescent antibody test (dFAT) and mouse intracerebral inoculation test (MIT). Exposed livestock recommendations included immediate vaccination, a strict isolation period of 90 days, and booster vaccinations during the third and eighth weeks.

**Conclusion:**

IHC revealed the widespread distribution of rabies virus antigen in the goat’s CNS, contrasting the discrete pathological changes. In this goat, definitive diagnosis of paralytic rabies was obtained through the association of epidemiological, clinical, laboratorial, pathological findings (histology and IHC) and gold standard confirmatory tests (dFAT and MIT).

## Background

High lethality, worldwide distribution and zoonotic potential make rabies a serious veterinary and public health problem, especially in developing countries [1, 2]. Therefore, accurate monitoring and diagnosis systems are of great importance since the correct identification of the infection has serious implications for post-exposure prophylaxis in humans and pre-exposure prophylaxis or euthanasia in animals [2, 3].

Paralytic form of rabies is frequent in cattle in Latin America [4–6], but it is uncommon in goats. In fact, there are few clinical reports on the furious form of rabies affecting goats [7, 8], and the sporadic cases of rabid goats from surveillance programs worldwide lack clinical data [9, 10]. Additionally, cerebrospinal fluid (CSF) analysis can be a useful tool for the diagnosis of neurological diseases, with few reports on CSF findings in rabid livestock [11]. Therefore, this report aims to describe epidemiological, clinical, laboratorial and pathological findings of paralytic rabies affecting a goat.

## Case presentation

Forty-seven Saanen goats of different ages were raised semi-extensively on a ranch in the municipality of Formosa, Goiás, Midwestern Brazil. During the day, the goats were released on a paddock for grazing native pasture; and at night, the animals were housed indoors. Flock management included free access to mineral supplementation and semiannual deworming. Animals did not receive any vaccines, and no vaccination protocols were implemented on the farm. The owner also stated the history of bat bites on the livestock on his ranch and surrounding farms. Six goats (mean age of two years) died within 15-days. Prior to spontaneous death, the goats presented apathy, isolation from the herd, sternal recumbence with self-auscultation position evolving to lateral recumbence with pedaling movements. Clinical evolution ranged from 3 to 5 days. These goats were buried by the owner; therefore, no laboratorial tests were performed.

According to the owner, a 1-year-old Saanen buck was isolated from the herd, characterizing the onset of clinical signs. At clinical evaluation the goat was alert, tachycardic (110 beats per minute), feverish (40.7 °C, 105.2 °F), dehydrated, and with discrete proprioceptive ataxia. No apparent skin lesions were present. Hematology parameters were within reference values [12]. Serum biochemistry profile showed slight hypoproteinemia (6 g/dL; normal range: 6.4–7 g/dL) by hypoalbuminemia (2.4 g/dL; normal range: 2.7–3.9 g/dL) [13], probably as consequence of anorexia. Due to the non-specific clinical signs, the goat received palliative treatment with intravenous fluids, dipyrone (Febrax™, Lemainjex, MG, Brazil: 20 mg.kg^− 1^, twice daily, intravenously) and oral vitamin supplementation.

**Fig. 1:**
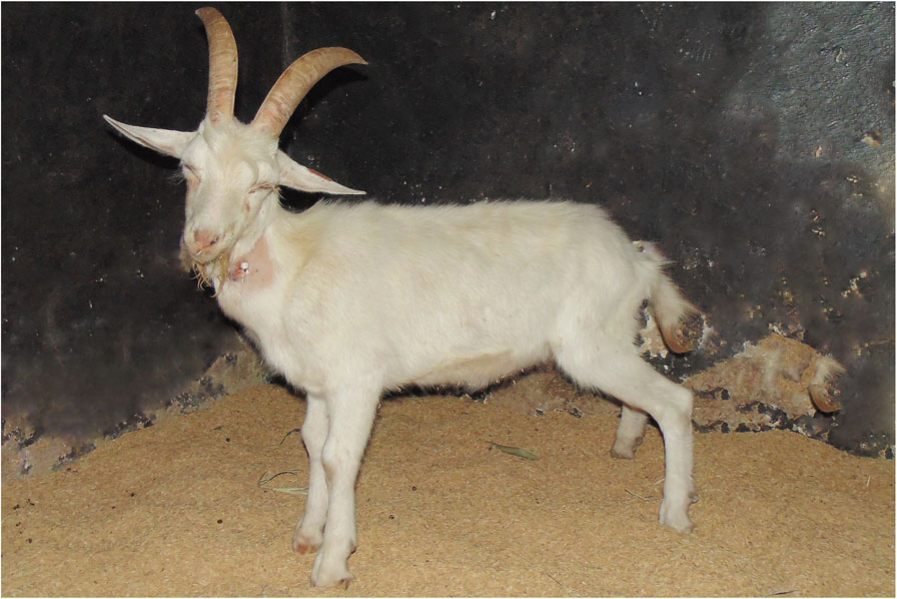
1-year-old Saanen buck presenting depression, somnolence and abnormal standing position

After 12-h evolution, the goat presented nervous signs that include depression, somnolence (Fig. 1), auricular hyperesthesia, opisthotonus episodes, and severe ataxia. Due to the onset of these signs, CSF analysis was performed; and presented mononuclear (69% lymphocytes, 25% monocytes, 5% neutrophils and 1% atypical lymphocytes) pleocytosis (29 nucleated cells/μL, normal range: 0-7 cells/μL), hyperproteinemia (46.7 mg/dL, normal range: 24–40 mg/dL) and high glucose levels (146 mg/dL, normal range: 45–87 mg/dL) [14]. On the CSF slide there was neither bacteria nor erythrophagocytosis or leukophagocytosis. A CSF sample was submitted to microbiological assays and resulted negative. Clinical evolution to lateral recumbence with pedaling movements and vocalization occurred within 8-h, totalizing 20-h of nervous evolution prior to euthanasia in extremis. Based on the association of epidemiological (bats bites and no rabies vaccination), clinical (brainstem, forebrain and medullary signs) and CSF analysis (mononuclear pleocytosis) findings, the presumptive diagnosis was rabies.

**Fig. 2:**
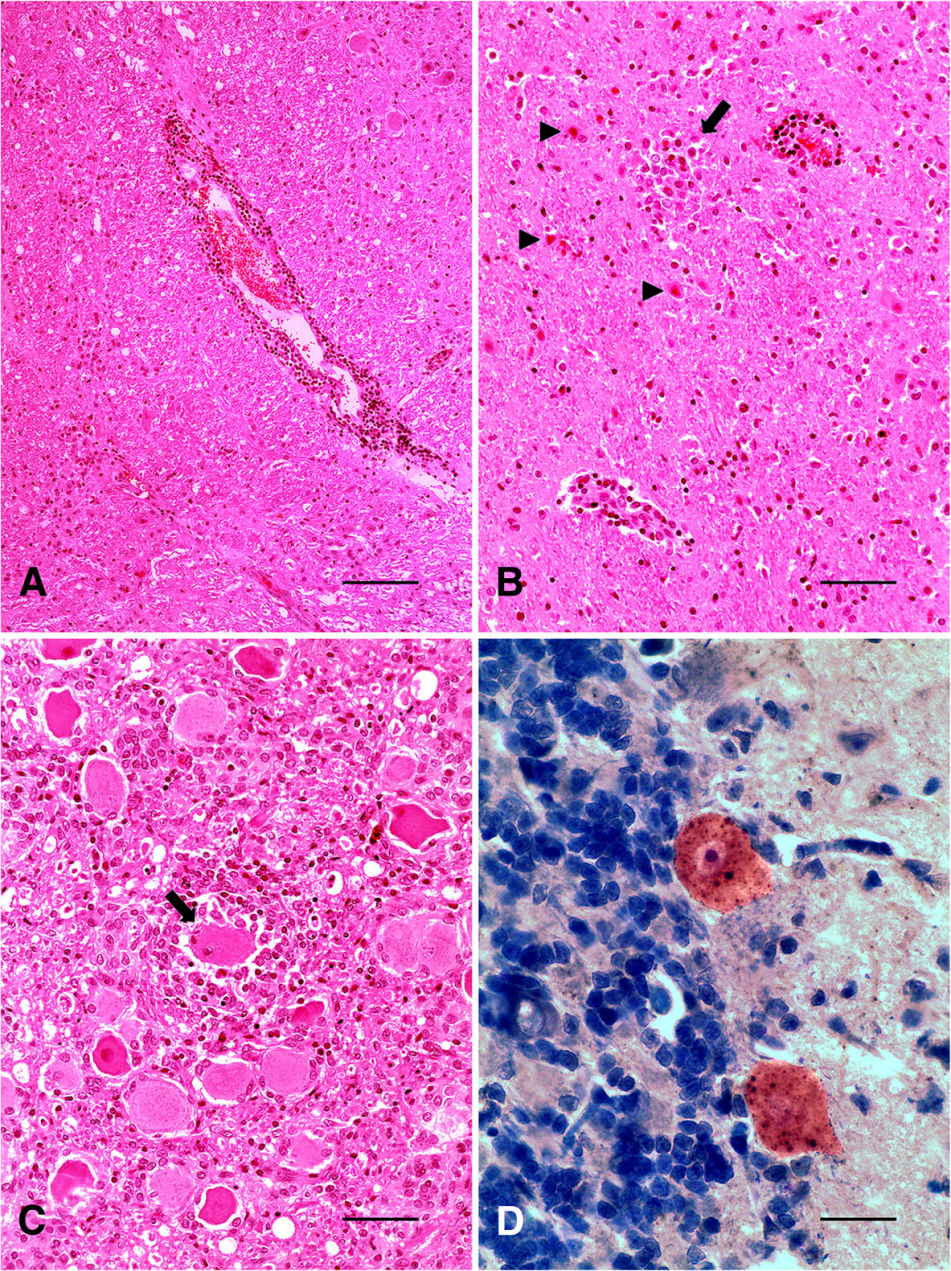
Goat. **a** Brainstem. Perivascular lymphoplasmacytic cuffing in white matter (H&E. bar = 100 μm). **b** Midbrain. Babès’ nodule (arrow) and necrotic neurons (arrow heads) at rostral colliculus (H&E. bar = 50 μm). **c** Gasser ´s ganglia. Neuronophagia (arrow) and mild mononuclear ganglioneuritis (H&E. bar = 50 μm). **d** Cerebellum. Anti-rabies virus immunolabeled Purkinje cells with strong reaction of the intracytoplasmic corpuscular structures (Streptavidin-peroxidase, chromogen 3,3-diaminobenzidine. Bar = 25 μm)

No gross lesions were observed at necropsy. Tissues samples were collected, fixed in 10% buffered formalin, embedded in paraffin and stained with hematoxylin and eosin (H&E). Central nervous system (CNS) fixed samples were submitted to immunohistochemistry (IHC) by the immunoperoxidase technique [1], and fresh samples sent for direct fluorescent antibody test (dFAT) and mouse intracerebral inoculation test (MIT) for the detection of rabies virus. Microscopically, main lesions were located in the brainstem and cervical spinal cord, consisting of discrete to moderate perivascular lymphoplasmacytic cuffing and multifocal hemorrhage areas in gray and white matter (Fig. 2a), and meninges. From the midbrain (rostral and caudal colliculus) to pons, small random multifocal glial nodules (Babès’ nodules) and necrotic neurons (Fig. 2b) were observed, surrounded by discrete astrocytosis. Gasser’s ganglia also showed small foci of neuronal necrosis, neuronophagia and mild mononuclear ganglioneuritis (Fig. 2c). Telencephalon, diencephalon and cerebellum presented discrete multifocal lymphoplasmacytic meningitis. Negri inclusion bodies (NI) were not observed in any of the evaluated regions of the CNS in H&E sections. Cerebellum, brainstem and cervical spinal cord demonstrated strong anti-rabies virus immunostaining of numerous small corpuscular intracytoplasmic structures in neurons (Fig. 2d). Fresh CNS samples were positive for the rabies virus in dFAT and MIT. CNS samples from a healthy goat was used as negative control for IHC, dFAT and MIT procedures.

After laboratorial rabies confirmation, all non-vaccinated person that handle the goat were submitted to post-exposure prophylaxis (PEP) composed of five vaccinations on days 0, 3, 7, 14, and 28 according to World Health Organization [15]. Recommendations for the exposed livestock consisted of immediate vaccination, a strict isolation period of 90 days, and booster vaccinations during the third and eighth weeks of the isolation period [16]. During these period, no other death was registered in the flock.

## Discussion

Rabies is an acute, progressive and fatal viral encephalitis caused by a neurotropic RNA virus in the family *Rhabdoviridae*, genus *Lyssavirus*, affecting all mammals [15]. In Europe and North America, most reported rabies is sylvatic, occurring primarily in wildlife reservoirs such as foxes, skunks, raccoons, and insectivorous bats [10]. In several parts of Asia and Africa, rabies remains urban, with dogs and cats comprising the majority of cases [7, 8]. In Latin America, rabies is an important problem, especially in dogs and cattle. Pets are the main source of human infection in urban areas, and vampire bats are considered the primary source of livestock infection [8, 17, 18]. Although rabies virus variant was not determined, it is possible to assume bats as an infection source but more data is needed to confirm this assumption, since mammalian wildlife as well as domestic carnivores are known to transmit rabies in Brazil [3, 8, 17].

Rabies in goats has been considered an infrequent disease [7], but regardless of the animal species affected, rabies is one of the most difficult diseases to diagnose clinically because of the diversity of potential presentations [7, 14], which are related to the inoculation site and subsequent neuronal spread of the virus [18]. Initially, the buck presented non-specific clinical signs (fever, dehydration and discrete proprioceptive ataxia); evolving to forebrain (somnolence and apathy), brainstem (proprioceptive ataxia and opisthotonus), and medullary (recumbence and pedaling movements) signs after a 20-h period. These features are compatible with paralytic rabies, presenting initially brainstem signs progressing to forebrain and medullary signs after viral spreading [18]. In goats, the furious form appears more commonly, and aggressive behavior may occur in 83% of the cases. Excessive bleating was observed in 72% of cases, salivation in 29%, and paralysis in only 17% [7]. These feature may be expected since most clinical reports on goat rabies are from Asia and Africa, where the canine rabies virus variant is the most prevalent [8]. The clinical course is usually between one and five days and always results in death [7, 14, 18]. Rabies laboratorial confirmation of the other six goats was impossible; but the shared epidemiological background, similar clinical signs and no new cases after proper PEP strongly suggests that this was a paralytic rabies outbreak.

CSF analysis is an integral component of the diagnostic evaluation of ruminants presenting with CNS signs [11, 18, 19]. Despite few reports on CSF findings in rabid ruminants, CSF analysis may be normal [14]. In this goat, it was an important ancillary tool because showed mononuclear pleocytosis (mainly lymphocytic), typically associated with viral infections [11]. Mild increase in protein concentration was also present and can be interpreted as a disruption of the blood-brain barrier [19], as observed in two cattle with rabies [11]. It has been suggested that CSF may act as a vehicle for the rapid rabies viral dissemination in the brain, but the virus can seldom be isolated from CSF [8]. Additionally, CSF analysis was important to allow exclusion of potential CNS bacterial infections that cause similar neurological signs, as meningoencephalitis, basilar empyema, brain abscesses, and listeriosis. Normally, CNS bacterial diseases presents relevant CSF abnormalities including turbid aspect, marked increase in protein concentration and neutrophilic pleocytosis [11, 19].

At necropsy, no significant lesions were observed. In rabies, characteristic macroscopic lesions are usually not reported and, when present, include non-specific changes such as hyperemia of leptomeninges and distension of the urinary bladder [2, 20]. Microscopically, non-suppurative meningoencephalitis is the main CNS lesion observed in all species, however may present variations in the anatomical distribution and intensity [9]. In the present case, lymphoplasmacytic meningoencephalitis was also detected in the goat; however, the absence of NI made diagnosis difficult, requiring ancillary tests for confirmation. It is important to note, despite the slight and non-specific microscopic changes and the absence of NI in the H&E stained CNS sections, IHC showed strong and widespread anti-rabies virus immunolabeling. dFAT and MIT were also positive and are considered the gold standard for diagnosis [15]. We reiterate rabies zoonotic potential, and suspected cases should be isolated and contact kept to a minimum with only vaccinated humans. Gloves and masks should be worn during examination, samples collection or when performing necropsies [7].

Inflammatory reaction in rabies is relatively mild, and may be more intense with focal hemorrhages in the brainstem and spinal cord [2, 15, 20], as detected at the microscopic evaluation. Additionally, NI presence and number appears to be inversely proportional to the degree of inflammation [20], which would be in agreement with the discrete inflammatory process observed in our case. Ruminants are considered highly susceptible to rabies infection and may show minor CNS inflammatory changes and a few or absent NI, especially in euthanized animals [18]. NI can be reliably detected only in 50–80% of infected animals [1, 20].

World Health Organization PEP recommendations in human exposed to rabies vary depending on the nature of the exposure and the vaccination status of the person exposed. Vaccination series is recommended for incidental contact with saliva, scratches, abrasions, or minor bites from suspect or confirmed rabid animals andmay be stopped if the animal is determined not to be rabid [15]. PEP of exposed livestock with a current vaccination status should be revaccinated immediately and observed for signs of rabies for a period of 45 days. Recommendations for PEP of unvaccinated livestock include immediate slaughter, or the aforementioned protocol performed on the goat flock from this report following the recommendations of Calan and Van Metre [16].

Clinical reports of rabies in goats are rare, and sometimes they are accompanied with atypical clinical-pathological presentations. Therefore, we reiterate the importance of its inclusion in the differential diagnoses of CNS diseases in goats. In this study, IHC revealed the widespread distribution of rabies virus antigen in the goat’s CNS, contrasting the discrete pathological changes. In this goat, definitive diagnosis of paralytic rabies was obtained through the association of epidemiological, clinical, laboratorial, pathological findings (histology and IHC) and gold standard confirmatory tests (dFAT and MIT).
